# Early-life adversity increases anxiety-like behavior and modifies synaptic protein expression in a region-specific manner

**DOI:** 10.3389/fnbeh.2022.1008556

**Published:** 2022-10-19

**Authors:** Jameel N. Hamdan, Jorge A. Sierra-Fonseca, Rodolfo J. Flores, Sigifredo Saucedo, Manuel Miranda-Arango, Laura E. O’Dell, Kristin L. Gosselink

**Affiliations:** ^1^Department of Biological Sciences, Border Biomedical Research Center, The University of Texas at El Paso, El Paso, TX, United States; ^2^Antharis Therapeutics, San Diego, CA, United States; ^3^Department of Science, Chatham University, Pittsburgh, PA, United States; ^4^Department of Psychology, The University of Texas at El Paso, El Paso, TX, United States; ^5^National Institutes of Health, National Institute of General Medical Sciences, Bethesda, MD, United States; ^6^Department of Physiology and Pathology, Burrell College of Osteopathic Medicine, Las Cruces, NM, United States

**Keywords:** stress, maternal separation, neuroplasticity, methamphetamine, addiction

## Abstract

Early-life adversity (ELA) can induce persistent neurological changes and increase the risk for developing affective or substance use disorders. Disruptions to the reward circuitry of the brain and pathways serving motivation and emotion have been implicated in the link between ELA and altered adult behavior. The molecular mechanisms that mediate the long-term effects of ELA, however, are not fully understood. We examined whether ELA in the form of neonatal maternal separation (MatSep) modifies behavior and synaptic protein expression in adults. We hypothesized that MatSep would affect dopaminergic and glutamatergic signaling and enhance sensitivity to methamphetamine (Meth) reward or increase anxiety. Male Wistar rats were subjected to MatSep for 180 min/d on postnatal days (PND) 2–14 and allowed to grow to adulthood (PND 60) with no further manipulation. The hippocampus (Hipp), medial prefrontal cortex (mPFC), nucleus accumbens (NAc), and caudate putamen (CPu) were isolated from one subgroup of animals and subjected to Western blot and protein quantitation for tyrosine hydroxylase (TH), α-synuclein (ALPHA), NMDA receptor (NMDAR), dopamine receptor-1 (D1) and –2 (D2), dopamine transporter (DAT), and postsynaptic density 95 (PSD95). Separate group of animals were tested for anxiety-like behavior and conditioned place preference (CPP) to Meth at 0.0, 0.1, and 1.0 mg/kg doses. MatSep rats displayed an increase in basal levels of anxiety-like behavior compared to control animals. MatSep rats also demonstrated CPP to Meth, but their responses did not differ significantly from controls at any drug dose. Increased NMDAR, D2, and ALPHA expression was observed in the NAc and CPu following MatSep; D2 and ALPHA levels were also elevated in the mPFC, along with DAT. MatSep rats had reduced D1 expression in the mPFC and Hipp, with the Hipp also showing a reduction in D2. Only the CPu showed elevated TH and decreased DAT expression levels. No significant changes were found in PSD95 expression in MatSep rats. In conclusion, ELA is associated with long-lasting and region-specific changes in synaptic protein expression that diminish dopamine neurotransmission and increase anxiety-like behavior in adults. These findings illustrate potential mechanisms through which ELA may increase vulnerability to stress-related illness.

## Introduction

Exposure to significant amounts of stress or adversity in early life can impact brain development and induce persistent neurological changes that may predispose an individual to mood, mental health, and substance use disorders in adulthood. Human and animal studies have investigated the effects of early-life adversity (ELA) on brain function and behavior and demonstrated that ELA increases the risk for both anxiety and addiction in adulthood (Huot et al., 2001; Sinha, 2008; Douglas et al., 2010; Hovens et al., 2012; Ishikawa et al., 2015). Similarly, accumulating evidence provides a strong link between adverse childhood experiences and the subsequent initiation of drug use, development of drug dependence, and increased risk for relapse in abstinent users (Dube et al., 2003; Shin et al., 2018; Derefinko et al., 2019). Stress, anxiety and substance abuse are thus highly interrelated but the neural mechanisms underlying the complex relationships between them are incompletely understood.

Brain regions and pathways that underlie motivation and reward have been implicated in the neurological and behavioral responses to ELA. At the same time, ELA has been shown to alter the function and regulation of these same structures and circuits (for review, see 23). The reward circuitry of the brain involves an interconnected network of dopaminergic, glutamatergic, and GABAergic signals acting at multiple forebrain sites including the nucleus accumbens (NAc), hippocampus (Hipp), medial prefrontal cortex (mPFC), and amygdala (Koob, 1992; Ikemoto and Panksepp, 1999). While dopamine release from the ventral tegmental area (VTA) to the NAc is a well-established component of reward, motivated behaviors are strongly influenced by GABA projections arising from the NAc. Moreover, an imbalance in communication within these circuits is highly associated with the development of mood disorders including anxiety. Lastly, the caudate putamen (CPu) serves as a terminal field for dopaminergic projections from the substantia nigra but has also been implicated in both goal-directed and habit learning (Seger and Spiering, 2011) and reward (Prado-Alcala and Wise, 1984).

Functional connections among neurons in these regions take time to develop. Prior investigations have shown that substantial time is needed for the development of VTA projections to the forebrain (Yetnikoff et al., 2014) and the dense, reciprocal connections between the amygdala and mPFC that do not fully establish until after adolescence (Hanson et al., 2021). The experience of ELA may therefore disrupt normal brain development, alter circuit connectivity, and induce long-lasting changes in neural function that may manifest as affective or substance use disorders. In support of this concept, recent studies have shown extensive and persistent effects of ELA on dopaminergic circuits in the prefrontal cortex, hypothalamus, and amygdala in adults (Gee et al., 2013), and significant influences of ELA on neurochemical pathways in the adolescent brain that predispose for drug dependence (Dimatelis et al., 2012).

The aim of the present work was to examine neurological responses to ELA that may contribute to increased anxiety or vulnerability to drug dependence in adulthood, and to test for relevant behavioral outcomes. Specifically, we assessed the effects of ELA in the form of maternal separation (MatSep) on changes in the expression of synaptic proteins involved in dopaminergic neurotransmission in reward-associated structures in the adult brain. We further assessed anxiety-like behavior and responses to methamphetamine (Meth) administration in adults. MatSep is a well-characterized stress model that can serve as an ELA stimulus (Anisman et al., 1998; Kuhn and Schanberg, 1998) and has been shown to impart long-term increases in anxiety-like behavior (Ishikawa et al., 2015) and drug-taking behavior (Lewis et al., 2013), as well as disruptions in cognitive performance (Eiland and McEwen, 2012). Our hypothesis was that ELA would alter synaptic protein expression in a manner that increases dopamine signaling and heightens both anxiety and sensitivity to drug reward. Thus, while the effects of ELA and, specifically, MatSep have been the subject of prior investigations, we aim to provide a better understanding of the synaptic mechanisms through which ELA might exert its effects and its long-term influence on mental health-related brain functions and behaviors.

## Materials and methods

### Experimental animals

Fully outbred Wistar rats were bred in-house and maintained in standard cages in a temperature- and humidity-controlled vivarium on a 12:12 h cycle with lights on at 0700 and food and water available *ad libitum*. All procedures were approved by the Institutional Animal Care and Use Committee of the University of Texas at El Paso and in accordance with the NIH Guide for the Care and Use of Laboratory Animals. Group sizes were determined using power analyses with effect size data from prior behavioral analyses of place conditioning, light/dark transfer, and open field testing in our laboratory.

### Early life adversity and behavioral testing groups

Neonatal MatSep was utilized as a form of ELA. On the day of their birth (postnatal day 0; PND 0), litters of pups were recorded but remained with their dams and undisturbed for PND 0–1. Pups were exposed to an early life adversity stressor in the form of maternal separation (MatSep) on PND 2–14 for 180 min/d between the hours of 0800 and 1,100. For this procedure, all pups from a single litter were transferred into a clean cage containing absorbent surgical bedding that was placed on top of a circulating water heating pad set at 37°C. The dams were removed from their home cage and individually placed in clean cages that were held in a vented biosafety cabinet across the room from the cages containing the pups. Following the separation period, the pups and the dams were returned to their original home cages. After the final separation period on PND 14, litters of pups remained with their dam continuously until PND 21 when they were weaned, separated by sex, and pair housed. Pups in control litters were gently handled on a daily basis during the PND 2–14 time window but were not separated from their dams at any point. Control and MatSep male rats used for this study were subsequently allowed to grow to adulthood (PND 60–65) without further manipulation. For all behavioral tests, the rats were randomly placed into different treatment conditions to avoid bias toward any given maternal offspring group. Observers were blind to the treatment condition. The ELA and control groups were derived from at least five different dam/sire pairings that produced 9–15 pups per litter. The rats were pair-housed with a same sex littermate from their respective treatment condition.

### Brain tissue harvest and processing

Adult male rats (*n* = 7–8/group) were lethally anesthetized (100 mg/kg pentobarbital, i.p.; Akorn Pharmaceuticals, Lake Forest, IL), and their brains rapidly harvested and frozen on dry ice. The NAc, Hipp, mPFC and CPu were isolated by gross dissection from 2 mm thick sections, collected according to the surface and deep anatomical landmarks illustrated in the Rat Brain Atlas of Paxinos and Watson (1998). Tissue samples were lysed by manual homogenization in tissue protein extraction reagent (T-PER) supplemented with protease and phosphatase inhibitors (Thermo Scientific, Waltham, MA), using a teflon-coated pestle in hand-held glass homogenizers. Sampling of Hipp and mPFC tissues was done bilaterally, while NAc samples were collected from the left hemisphere only. Lysates were centrifuged at 10,000 × *g* for 10 min and the cleared lysate subjected to protein quantitation using the Pierce bicinchoninic acid assay (ThermoFisher Scientific, Rockford, IL, USA) with bovine serum albumin (BSA) as a standard. Protein samples were further subjected to SDS-PAGE and Western blotting as described below.

### Antibodies

The primary antibodies, sources, and dilutions are described in Supplementary Table 1. Alkaline phosphatase (AP)-conjugated secondary antibodies (goat anti-rabbit and goat anti-mouse) were obtained from Southern Biotech (Birmingham, AL) and used at a 1:200 dilution.

### Electrophoresis and Western blotting

Twenty micrograms of total protein from each sample were subjected to SDS-PAGE on pre-cast Criterion^®^ 10–20% gradient polyacrylamide gels (Bio Rad, Hercules, CA, USA) and run at 80V for 20 min to stack the proteins, then 140V for 90 min to allow protein migration through the gel. Proteins were then transferred to PVDF membranes (14 h at 20V, 4°C) (BioRad, Hercules, CA, USA) and blocked for 1 h at room temperature with 1% BSA in Tris-buffered saline (10 mM Tris, pH 7.4) with 0.05% Tween-20 (TBST; Sigma Aldrich, St. Louis, MO, USA). Primary antibodies were pre-diluted in blocking buffer and applied to the membranes, then incubated overnight at 4°C. Actin was used as a loading control. Membranes were washed in TBST and incubated for 1 h at room temperature in AP-conjugated secondary antibodies (goat anti-rabbit or anti-mouse) diluted in blocking buffer. For all blots, Precision Plus Protein Dual Color Standards (BioRad, Hercules, CA) served as a molecular weight standard.

### Densitometric analysis

Protein bands were detected by incubation of the membranes in Immun-Star chemiluminescent reagent (BioRad, Hercules, CA, USA) for 5 min, followed by exposure to autoradiographic film (Thermo Scientific, Waltham, MA) for 2 min. Signal intensity was analyzed by densitometry using LabWorks software (UVP Laboratory Products, Upland, CA, USA). In some experiments, the chemiluminescent signal was directly imaged and quantitated using the iBright FL1500 imaging system (Thermo Scientific, Waltham, MA, USA).

### Behavioral testing

#### Open field

A subset of adult male MatSep and control rats (*n* = 6/group) was tested for anxiety-like behavior in an open field test. A Plexiglas^®^ apparatus with opaque walls and a clear lid was placed on a flat surface in a quiet, well-lit room. The floor of the apparatus was divided into a grid of 36 squares. Experimenters placed individual rodents into the field and recorded their behavior for 5 min (300 s). The apparatus was thoroughly cleaned after each animal. Blinded observers viewed the video recordings and documented animal behaviors including defecation, rearing, grooming, freezing, locomotion, and time spent in the peripheral areas compared to the center of the grid.

#### Light/dark transfer

Further assessment of anxiety-like behavior was done in a subset of control and MatSep animals (*n* = 5/group) prior to their entry into the conditioned place preference paradigm (see below), using a light/dark transfer box. The apparatus employed in this study consisted of two side-by-side enclosed chambers, one with clear Plexiglas^®^ walls and another one with solid black walls, and a small connecting door between them. At the beginning of the test, the rats were placed into the dark side of the apparatus and were given free access to both light and dark areas. The time spent in each side was recorded for 5 min. An increase in anxiety-like behavior in MatSep animals was defined as a significant decrease in the amount of time spent in the light portion of the chamber compared to the time observed in controls. The number of transitions between the dark and light sides of the chamber was also quantified.

#### Conditioned place preference

The responses of control and MatSep animals to the rewarding effects of Meth were examined in a conditioned place preference (CPP) paradigm. Our conditioning chambers consisted of 2 distinct compartments of equal proportions (76 × 24 × 30 cm) that were separated by a removable solid partition. One compartment had black walls with pine bedding beneath a smooth Plexiglas^®^ floor with small holes. The other compartment had black and white striped walls with a mixture of pine bedding and blue paper chips beneath a textured Plexiglas^®^ floor that also contained small holes. Both compartments were equally illuminated, and continuous white noise (0–20 kHz) was used to minimize any disturbances from outside the test area. This study employed a biased procedure that consisted of three phases: an initial pre-test, 8 days of conditioning, and a final post-test. Our experimental parameters were based on prior work with nicotine and a biased conditioning design was used because these procedures have been shown to be more sensitive at detecting shifts in the rewarding effects of drugs of abuse (O’Dell and Khroyan, 2009). To test for preference behavior, the solid partition that separated the compartments was removed and replaced with a partition that had an opening in the center (8 × 8 cm high). The rats were allowed to shuttle freely between the compartments for 15 min. In our experience, the rats generally prefer the solid black chamber, and we eliminate rats that display an initial bias of greater than 65% because it is difficult to establish CPP in rats that display a strong initial preference for either compartment. Five days after the initial pre-test, an 8-day conditioning procedure was initiated using 4 drug pairings and 4 saline pairings in 30-min sessions. The day after the last conditioning session, the rats were re-tested for shifts in preference behavior for 15 min. In each study, the order of drug treatment was counterbalanced within treatment groups such that some rats received drug on the first day of conditioning and the other half of rats received drug on the second day of conditioning. The animals were conditioned using different doses of Meth (Millipore Sigma, St. Louis, MO, USA) *via* repeated i.p. injections of either 0, 0.1, or 1.0 mg/kg doses of the drug. The post-test was conducted on PND 83. A positive CPP result was defined as a significant increase in time spent in the drug-paired chamber during the post-test versus to the pre-test.

### Statistical analyses

#### Densitometry

For all Western blot experiments, individual samples were run in triplicate and their densitometry scores normalized to actin expression and averaged. Data points > 1.5 standard deviations from the group mean were removed from the study. Protein expression levels from control animals in each brain region were set to 100%, and the data from MatSep animals are presented as percent of control ± standard error of the mean (SEM). Results were compared by t-test, with *p* ≤ 0.05 considered statistically significant.

#### Behavioral testing

For the CPP test, the% time spent in the non-preferred the preferred side was calculated as a function of the total 15 min test period, and these values were calculated separately during the pre-test before conditioning and during the post-test after the conditioning sessions were completed. The data were analyzed by comparing group differences between drug-paired rats and their respective controls during the pre- and post-test separately. Also, we compared within-subject changes in time spent in the drug-paired (initially non-preferred) side before and after conditioning in each treatment group. Locomotor activity was also evaluated by counting the number of midline crosses made by the rats between the two chambers. Difference scores and midline crosses were compared using a two-way ANOVA with methamphetamine dose and treatment group (MatSep vs. control) as between-subject factors and Tukey *post hoc* analysis, with *p* ≤ 0.05 considered statistically significant.

For analysis of anxiety-like behavior in the light/dark transfer box, the percent time spent in the light side of the apparatus was compared between MatSep and control rats. Also quantified was the number of transitions made between light and dark areas. For the open field analysis, simple counts of behaviors displayed or the amount of time they were displayed were recorded. Results were compared across groups by *t*-test, with *p* ≤ 0.05 indicating statistical significance. For all behavioral tests, rats were eliminated from the analysis if their values exceeded 1.5 standard deviations from the group mean.

## Results

In the present study, exposure to MatSep was found to alter protein levels in a region-specific fashion in the brains of adult male rats as indicated by Western blot analysis. This model of ELA, a neonatal stress paradigm, affected synaptic protein expression in the Hipp, mPFC, NAc and CPu. A complete set of blot images is provided in Supplementary material. Behavioral changes were also observed in adults with prior exposure to MatSep, as these animals demonstrated increased anxiety-like behavior in the light/dark transfer and open field tests.

### Region-specific changes in the expression of synaptic proteins

**FIGURE 1:**
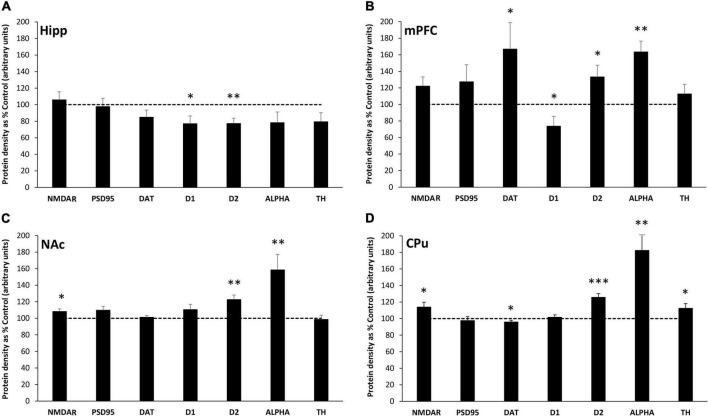
MatSep modifies synaptic protein expression in the Hipp **(A)**, mPFC **(B)**, NAc **(C)** and CPu **(D)**. Densitometric analysis of synaptic protein expression, normalized to actin and averaged over at least 3 separate blots, is presented from MatSep animals (*n* = 7–8/group) expressed as% control (mean ± SEM). The dashed line indicates the 100% control level of expression. Statistically significant differences between MatSep and Control are designated by: *p ≤ 0.05; ^**^p ≤ 0.01, ^***^p ≤ 0.001. NMDAR, N-methyl-D-aspartate (NMDA) receptor; PSD95, postsynaptic density 95 protein; DAT, dopamine transporter; D1, dopamine receptor-1; D2, dopamine receptor-2; ALPHA, α-synuclein; and TH, tyrosine hydroxylase.

The effect of MatSep on the expression of synaptic proteins in the Hipp, mPFC, NAc and CPu of adult male rats was measured by Western blotting. In the Hipp, exposure to MatSep was associated with significant decreases in D_1_ (*p* ≤ 0.05) and D_2_ (*p* ≤ 0.01) receptor expression compared to the levels observed in controls (Figure 1A). Non-significant trends toward reduced expression were also seen for the DAT (*p* = 0.06) and TH (*p* = 0.06) in the Hipp. Significant changes in protein expression were also observed in the mPFC of MatSep rats compared to control animals (Figure 1B), including increases in D_2_ (*p* ≤ 0.05) and ALPHA (*p* ≤ 0.01). DAT expression was also markedly elevated (*p* ≤ 0.05) in this region, in contrast to the other brain areas examined, while D_1_ levels were significantly decreased (*p* ≤ 0.05). There was a non-significant trend (*p* = 0.07) toward increased NMDAR expression in the MatSep mPFC. In the NAc, MatSep significantly increased D_2_ and ALPHA levels (*p* ≤ 0.01), similar to the results seen in the mPFC (Figure 1C). NMDAR expression was also significantly increased in this region (*p* ≤ 0.05), while the expression of PSD95, DAT, D_1_, and TH remained unchanged (*p* > 0.05). There was a non-significant trend (*p* = 0.07) toward increased PSD95 expression in the NAc, however. As was observed in the mPFC and NAc, D_2_ and ALPHA protein levels were elevated in the CPu of MatSep animals compared to controls (Figure 1D; *p* ≤ 0.001 and *p* ≤ 0.01, respectively). Increased NMDAR and decreased DAT expression was also seen in this region (*p* ≤ 0.05), and the CPu was the only site evaluated in which increased TH expression wa *p* s seen (*p* ≤ 0.05). A summary of all changes in protein expression that were seen in response to MatSep is provided in Table 1.

**TABLE 1:** Significant changes in synaptic protein expression induced by MatSep in adult male rats.

Marker	Change with MatSep
NMDAR	↑ in NAc
*PSD95*	*(none)*
DAT	↑ in mPFC
D1	↓ in Hipp and mPFC
D2	↑ in NAc and mPFC; ↓ in Hipp
ALPHA	↑ in NAc and mPFC
*TH*	*(none)*

↑, increased expression; ↓, decreased expression.

### Conditioned place preference and light/dark transfer behavioral tests

**FIGURE 2:**
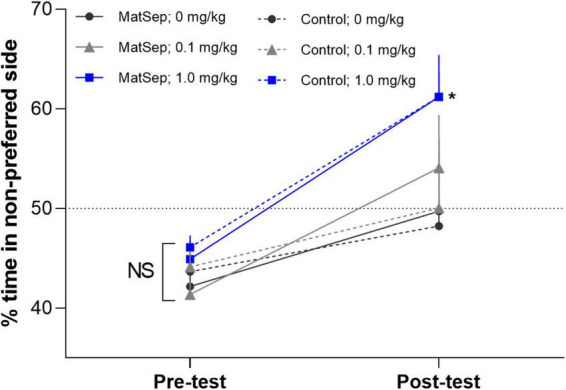
MatSep has no effect on CPP to Meth. The data reflect% time spent in the non-preferred side as a function of the total 15 min test period. These values were calculated during the pre-test before conditioning and during the post-test after the conditioning. The magnitude of CPP was compared in control and MatSep rats (*n* = 5–8/group) before (pre-test) and after (post-test) conditioning with 3 doses of Meth (0, 0.1, or 1.0 mg/kg, i.p.). The results revealed that CPP is produced by administration of the highest dose of Meth (*p ≤ 0.05), but MatSep has no additional effect on this response. The dotted line at 50% demarcates where there is no shift in preference behavior.

**FIGURE 3:**
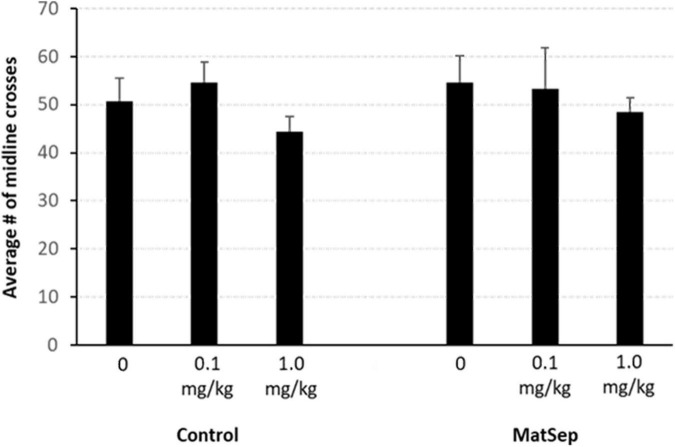
Locomotor activity is not affected by MatSep or Meth administration. Locomotor activity was quantified as the number of times an animal crossed the midline of the CPP apparatus, in transition from one chamber to the other. No significant differences were seen, regardless of treatment group or drug dose used for conditioning (*n* = 5–8/group).

An analysis of the CPP data, revealed that there were no group differences in% time spent in the non-preferred side during the pre-test (Figure 2). However, during the post-test, there was an overall difference across treatment groups [*F*(2,34) = 7.725, *p* = 0.002; partial η^2^ value = 0.312]. A within subject analysis of pre- versus post-test values, revealed that the highest dose of Meth (1.0 mg/kg) produced a significant shift in% time spent in the non-preferred side that was similar in control (*p* = 0.012; Cohen’s *d* effect size value = 1.328) and MatSep (*p* = 0.0001; Cohen’s *d* effect size value = 2.703) treatment groups. Administration of the lowest dose of Meth (0.1 mg/kg) did not produce a significant shift in pre- versus post-test values in either control (*p* = 0.998; Cohen’s d effect size value = 0.368) or MatSep (*p* = 0.230 Cohen’s d effect size value = 0.862) treatment groups. Locomotor activity was also assessed during the post-test, as the number of times an animal crossed the midline in transition from one chamber of the CPP apparatus to the other (Figure 3). No significant differences were seen between groups with either the 0.1 or 1.0 or mg/kg dose [*F*(2,34) = 0.535, *p* = 0.591; partial η^2^ value = 0.305], no differences were observed between MatSep and control animals [*F*(1,34) = 0.00, *p* = 1.0; partial η^2^ value = 0), and no interaction between dose and treatment group was indicated [*F*(2,34) = 0.00, *p* = 1.0; partial η^2^ value = 0)].

**FIGURE 4:**
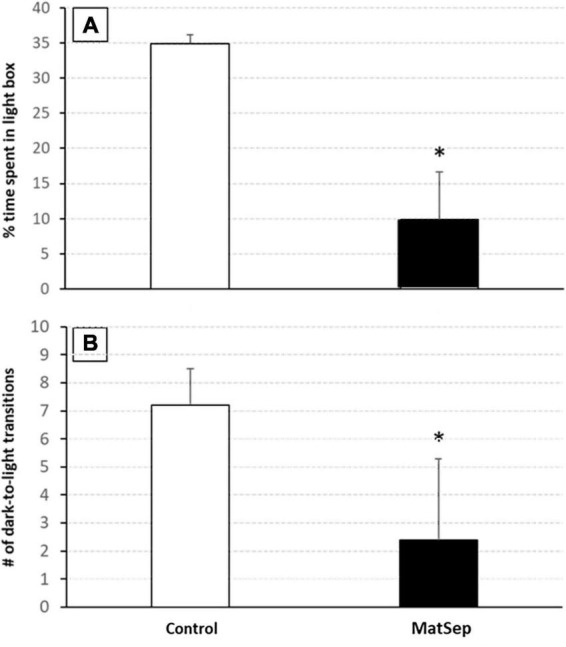
Anxiety-like behavior is increased in MatSep adults. Exposure to MatSep in the neonatal period is associated with increased anxiety-like behavior in adulthood, as evidenced by a decreased amount of time spent in the light portion of the light/dark box **(A)**, and a reduced number of transitions made from the dark to the light areas of the testing apparatus **(B)** demonstrated by MatSep animals. *p ≤ 0.05; *n* = 5/group.

In the light/dark transfer test, significant increases in anxiety-like behavior were observed in MatSep animals compared to controls (Figure 4). The increase in anxiety is represented by a reduced amount of time spent in the light side of the light/dark apparatus (Figure 4A; *p* = 0.005), and further supported by a diminished number of transitions made from the dark to the light environment (Figure 4B, *p* = 0.009). Additional testing for anxiety-like behavior was done in an open field setting, the results of which are shown in Table 2. Compared to control animals, adult males with prior exposure to MatSep demonstrated significant reductions (*p* ≤ 0.05) in grooming behavior in terms of both number of grooming episodes and the amount of time spent grooming, and decreased locomotion as evaluated by the number of gridlines crossed during the testing period.

**TABLE 2:** Maternal separation increases some anxiety-like behaviors in adult male rats subjected to an open field test.

Treatment	Number of defecations	Number of rearings	Rearing time (s)	Number of groomings	Grooming time (s)	Number of gridlines crossed	Number of center entries	Center time (s)	Periphery time (s)
Control	1.3 ± 0.88	45.5 ± 5.18	88.5 ± 6.96	1.5 ± 0.22	11.5 ± 2.79	258.5 ± 10.80	4.8 ± 1.30	8.2 ± 1.45	291.7 ± 1.54
MatSep	0.3 ± 0.33	54.2 ± 6.12	91.3 ± 5.99	0.5 ± 0.22*	2.8 ± 1.28*	210.8 ± 17.47*	3.3 ± 1.23	5.5 ± 1.93	294.5 ± 1.93

Data are presented as mean ± SEM (n = 6/group). *Significant difference (p ≤ 0.05) between Control and MatSep.

## Discussion

### Changes in protein expression induced by maternal separation

The present study revealed MatSep-induced alterations in the expression of protein markers involved in neurotransmission in the NAc, a brain region that plays a central role in the reward pathways associated with drug use and dependence (Koob and Bloom, 1988; Koob and Volkow, 2010). Increased levels of D_2_, ALPHA, and NMDAR expression were observed, indicating altered synaptic function in this region in adult male rats that were exposed to ELA as neonates. Moreover, both pre-and post-synaptic mechanisms appear to be altered in these animals, which may result in an overall reduction in dopamine neurotransmission. Complex roles have been described for D_2_, which localizes to both sides of the synapse and can inhibit dopamine synthesis or the depolarization of postsynaptic membranes through its activation (De Mei et al., 2009). An increase in D_2_ expression, therefore, is likely to decrease dopamine transmission in the NAc although we are unable to precisely determine the pre- or post-synaptic site of this effect. ALPHA is a presynaptic regulator of vesicular trafficking that reduces neurotransmitter secretion, and the increased expression of this protein seen in our MatSep animals should also limit basal dopamine release to the NAc. Interestingly, ALPHA has been shown to play an important role in the decreased dopamine release seen in rats with a high-anxiety phenotype (Rocchetti et al., 2015). In apparent contrast to our findings, another recent investigation showed that ELA increases excitability in dopaminergic neurons of the VTA and enhances downstream dopamine release in the NAc (Shepard et al., 2020). The subjects of this work were juvenile rats, however, and their MatSep paradigm differed from the one we employed. Our results support the interpretation that basal dopaminergic neurotransmission is reduced in adult male rats with a history of ELA.

Given the importance of dopamine in drug use and dependence, our findings indicate an influence of ELA on the development of these conditions, possibly even a protective effect. Alternatively, however, the markedly elevated ALPHA expression observed in this study may promote an increase in the available pool of neurotransmitter and enhance dopamine release in the presence of drug. This has been shown in animals over-expressing ALPHA and given access to cocaine (Boyer and Dreyer, 2007), indicating that high ALPHA levels in the MatSep NAc may increase vulnerability to addiction. Behavioral consequences of cocaine intake (Pulvirenti et al., 1992) or increased NAc dopamine release (Kelley and Throne, 1992) are mediated, at least in part, by the NMDAR which was also elevated in the NAc of ELA rats in our study. Reduced glutamatergic neurotransmission has been seen following a different form of ELA, in which an environment with limited bedding and nesting materials led to reduced glutamate levels (Ordoñes Sanchez et al., 2021) and decreased AMPA receptor expression (Ganguly et al., 2019) in the NAc of adult male rats. Since glutamate signaling and subsequent neuroplasticity are governed by the AMPA:NMDA receptor ratio (Hearing et al., 2016), changes in the expression of these proteins can lead to marked functional alterations. We did not assess AMPA receptor expression in the NAc, but the increase in NMDAR expression in the present study suggests an imbalance in glutamatergic inputs to the NAc in adult rats with prior ELA exposure in the form of MatSep. As addiction and an increased risk for relapse are both associated with impaired glutamate homeostasis in the NAc (Zhang et al., 2021), this may be another mechanism through which vulnerability to addiction is increased following ELA.

In the Hipp, which provides glutamatergic inputs to the NAc and plays a major role in cognitive function, evidence of reduced dopamine signaling was also observed in our ELA animals. Decreases in both D_1_ and D_2_ expression were observed in this region, along with a non-significant (*p* = 0.06) trend toward a decrease in TH levels. These changes would limit dopamine synthesis and action, an effect that may be partially compensated for by the suggestive increase in DAT expression that was also seen but did not reach significance (*p* = 0.06). In line with our findings, some aspects of cognitive dysfunction have been associated with abnormal dopaminergic neurotransmission and diminished DAT expression in the Hipp (Borgkvist et al., 2012). Moreover, a role for D_2_ has been identified in both the presynaptic and postsynaptic domains as D_2_ antagonist administration reduces long-term potentiation in the Hipp but removal of this protein from the presynaptic membrane only decreases long-term depression and impairs spatial memory (Rocchetti et al., 2015). A recent study (Holubová et al., 2018) found that exposure to chronic postnatal stress in the form of MatSep led to declines in cognitive function in male rats, and this effect persisted into adulthood. It has also been shown that basal performance on Hipp-dependent memory tasks is impaired in MatSep male rats tested as adults, suggesting blunted plasticity in this region (Eiland and McEwen, 2012). Altering these basic functional mechanisms within the Hipp could have far-ranging impacts on the brain and behavior, contributing to numerous conditions including affective disorders and increased vulnerability to addiction.

Numerous connections have been drawn between Hipp function and the experience of stress, indicating a complex and multifactorial relationship through which stress can modify cognitive performance and behavior. The Hipp is sensitive to a broad range of circulating glucocorticoid levels and tends to inhibit HPA axis activity through glutamatergic activation of local hypothalamic GABA circuits (Jankord and Herman, 2008). Stress-induced elevations in corticotropin-releasing hormone (CRH) secretion lead to increased glucocorticoid release which mediates systemic stress responses. At the same time, the hypersecretion of CRH during ELA can induce long-lasting behavioral and neuroendocrine changes that underlie the later development of anxiety disorders (Binder and Nemeroff, 2010). CRH synthesis and signaling within the Hipp itself plays a critical role in how stress impairs learning and memory (Hooper et al., 2018), and the overexpression of CRH in postnatal rats has been shown to decrease D_1_ and D_2_ receptor expression and disrupt dopamine signaling in the Hipp (Kasahara et al., 2011), similar to the effects seen in the present study.

Increased expression of the D_2_, DAT, and ALPHA proteins was seen in the mPFC of adult male rats exposed to ELA in the form of neonatal MatSep. With substantial contributions to regulating attention, cognition, and memory, this brain region connects to other limbic and reward structures and has been implicated in inhibitory control mechanisms associated with goal-directed behaviors (Gusnard et al., 2001). A concurrent decrease in D_1_ expression was also observed, enhancing the relative role of D_2_ and shifting the balance of synaptic effects in favor of this receptor which tends to reduce dopaminergic signaling as previously described. Further decreases in dopamine availability are likely to result from the elevated DAT and ALPHA levels seen after ELA exposure, which may limit the ability of the mPFC to effectively regulate its targets. In humans (Gee et al., 2013) and rodents (Ishikawa et al., 2015), exposure to ELA has been shown to reduce the functional connectivity between the mPFC and amygdala, leading to an early maturation of this circuit and an increased expression of anxiety or anxiety-like behavior. These brain regions are also strongly implicated in drug use behavior (Koob and Volkow, 2010), as well as the loss of control of drug intake that accompanies the transition to dependence (Goldstein and Volkow, 2011). Thus, the changes in mPFC synaptic protein expression levels seen in the present study may underlie an increased vulnerability in our MatSep rats to the development of affective or substance use disorders.

Dopamine in the CPu is heavily involved in the planning and control of motor function, and has been implicated in motivated behavior and reward. MatSep significantly impacted the expression of multiple proteins involved in dopaminergic neurotransmission in this brain region in adults, the consequences of which require further investigation. Increased levels of D_2_ and ALPHA, as discussed previously, support a condition of reduced synaptic release. At the same time, the increased TH and decreased DAT expression we observed could be engaged as compensatory mechanisms to maintain appropriate levels of dopamine at the synapse. The CPu was, in fact, the only brain area investigated in which an elevated expression of TH was seen. This is in line with earlier work that identified a high proportion of membrane-bound TH in the CPu that may be able to provide a unique or rapidly-synthesized pool of dopamine or other catecholamines (Segal and Kuczenski, 1974). The complex neural circuitry that underlies motor behavior includes the direct and indirect pathways that are activated by the CPu. Binding of dopamine to D_1_ receptors leads to direct pathway activity, while D_2_ receptor binding subsequently inhibits the indirect pathway (Self and Nestler, 1995). The increased expression of D_2_ receptor found in this study, therefore, suggests an increased inhibition of the indirect pathway. While no changes in D_1_ expression were seen, the elevated levels of NMDAR observed could again suggest an imbalance in dopamine:glutamate signaling and the potential for dimerization of these two receptor types could functionally enhance the effects of glutamate (Cepeda et al., 1993).

### Maternal separation increases baseline levels of anxiety but not conditioned place preference

In behavioral testing using light-dark transfer and open field tests, we demonstrate that adult rats who were exposed to ELA through neonatal MatSep display increased basal levels of anxiety-like behavior. This is consistent with previous findings in which MatSep rats showed higher anxiety when tested as adolescents (Jin et al., 2018) or adults (Huot et al., 2001), and human studies demonstrating that children experiencing parental neglect (Hovens et al., 2012) or separation from one or both parents (Kendler et al., 1992; Bandelow et al., 2004) have a significantly increased risk of developing anxiety in adulthood. One of these research teams, in fact, identified separation experiences and family history as the most critical factors in the later development of social anxiety disorder (Bandelow et al., 2004). Our ELA model in which pups were separated from their dam for 3 h/d is well-established as a stress paradigm that frequently induces long-lasting neurochemical and behavioral effects. In other studies, however, MatSep has led to increased resilience in the pups (Rana et al., 2015), especially if the separation takes place over a shorter (e.g., 15 min) period (Alizadeh-Ezdini and Vatanparast, 2022).

Parental separation or neglect have also been identified as causative factors in the development of substance use disorders in adults (Dube et al., 2003; Räikkönen et al., 2011; Norman et al., 2012). As such, we expected to see increased sensitivity to Meth in rats with prior exposure to ELA. Under the current experimental conditions, however, MatSep rats displayed CPP to a 1.0 mg/kg of Meth that was equivalent to that seen in control animals. Locomotor activity also did not differ between groups in this behavioral paradigm. Similar findings have been published previously (Faure et al., 2009; Hensleigh and Pritchard, 2014; Womersley et al., 2016), in which MatSep and non-stressed rats showed similar CPP responses to Meth at this dose. In another study, however, MatSep rats demonstrated increased Meth self-administration behavior and a more rapid escalation of Meth intake compared to controls (Lewis et al., 2013), leading us to investigate whether increased Meth reward sensitivity might manifest as a CPP response to a low dose of drug following ELA. Contrary to our hypothesis, control and MatSep animals showed equivalent behavior and both groups failed to establish CPP at a lower (0.1 mg/kg) dose of Meth. That the CPP responses of the MatSep animals did approach significance, however, suggests the possibility that CPP to a low Meth dose may be achievable with longer or more frequent exposure. A few studies have shown increased CPP and sensitivity to the rewarding effects of other drugs including alcohol (Leichtweis et al., 2020) and nicotine (Dalaveri et al., 2017) in MatSep rats, but these investigations were carried out in adolescent rats. Investigations into cocaine self-administration by MatSep rats, however, have produced variable results with one group (Matthews et al., 1999) showing diminished intake and another (Moffett et al., 2006) demonstrating acquisition at lower drug doses. In another study, ELA in the form of neonatal MatSep appeared to increase the rewarding effects of amphetamine and the risk for developing addiction, but only in rats challenged with a proximal stressor in adulthood (Der-Avakian and Markou, 2010). Lastly, CPP involves drug administration rather than voluntary drug seeking or consumption and may thus rely on opioid systems to a greater degree than dopaminergic pathways. As neonatal handling paradigms have been shown to persistently alter opioid receptor expression (Ploj and Nylander, 2003; Kiosterakis et al., 2009), future work should address this concept. Together, results from the field clearly identify a role for ELA in drug use and addiction, but the present study further demonstrates that variable responses can emerge with the use of different substances and scenarios.

## Conclusion

Our results demonstrate that ELA in the form of neonatal MatSep is associated with region-specific changes in synaptic protein expression that persist into adulthood. These changes may alter dopaminergic and other forms of neurotransmission and serve as mechanisms through which long-term behavioral changes manifest because of stress experienced in early life. Indeed, the increased anxiety-like behavior observed in our animals shows that the impacts of ELA can be long-lasting and render individuals more vulnerable to developing affective and other disorders at times far distant from when the initial stressor was experienced. Future studies would benefit from examining the role of the amygdala in ELA-associated brain and behavioral changes. Future work might also include the examination of sex differences, as females may be particularly vulnerable to the long-term effects of ELA (Eck et al., 2020). Interestingly, in a small pilot study by our laboratory, adult female rats exposed to MatSep as neonates did not display the same anxiety-like behaviors in the open field test that were observed in the males reported here (data not shown). The contributions of oxytocin signaling to stress-related increases in addiction vulnerability is also an important question as recently described (Bardo et al., 2021). Our work, however, advances our understanding of how synaptic function can be significantly altered for extended periods of time in response to ELA and may predispose an individual to conditions of diminished mental health.

## Data availability statement

The original contributions presented in this study are included in the article/Supplementary material, further inquiries can be directed to the corresponding author.

## Ethics statement

The animal study was reviewed and approved by IACUC, The University of Texas at El Paso.

## Author contributions

JH, LO’D, and KG conceived and designed the study. JH, JS-F, and KG wrote the manuscript. All authors were involved in data acquisition, analysis, interpretation, read, revised, and approved the final manuscript.
