# Supplementary material for: Early-life adversity increases anxiety-like behavior and modifies synaptic protein expression in a region-specific manner
Source: Front Behav Neurosci. 2022 Oct 19;16:1008556. doi: 10.3389/fnbeh.2022.1008556 (PMC9626971; doi:10.3389/fnbeh.2022.1008556)
Supplement: Supplementary file 1 [file Table_1.DOCX]

Supplementary Material

**Supplementary Table S1.** Primary and secondary antibodies used in Western blotting.

| **Antibody** | **Dilution** | **Supplier** | **Catalog #** |
| --- | --- | --- | --- |
| RB anti-D_1_ receptor | 1:1000 | Abcam, Cambridge | ab81296 |
| RB anti-D_2_ receptor | 1:1000 | EMD Millipore, Burlington, MA | AB5084P |
| RB anti-DAT (H-80) | 1:1000 | Santa Cruz Biotechnology, Dallas, TX | sc14002 |
| RB anti-PSD-95 (7E3) | 1:1000 | Cell Signaling Technology, Danvers, MA | 36233p |
| RB anti-α-synuclein | 1:1000 | Cell Signaling Technology, Danvers, MA | 2628s |
| RB anti-NMDA NR1 receptor (variant N1) | 1:1000 | R&D Systems, Minneapolis, MN | PPS083 |
| MS anti-TH (clone LNC1) | 1:1000 | Millipore, Burlington, MA | MAB318 |
| MS anti-Actin | 1:2000 | Invitrogen, Carlsbad, CA | MA5-11869 |

RB, rabbit; MS, mouse.
