# Supplementary figures and images for: Early-life adversity increases anxiety-like behavior and modifies synaptic protein expression in a region-specific manner
Source: Front Behav Neurosci. 2022 Oct 19;16:1008556. doi: 10.3389/fnbeh.2022.1008556 (PMC9626971; doi:10.3389/fnbeh.2022.1008556)

Supplementary Material – full Western blot images

#
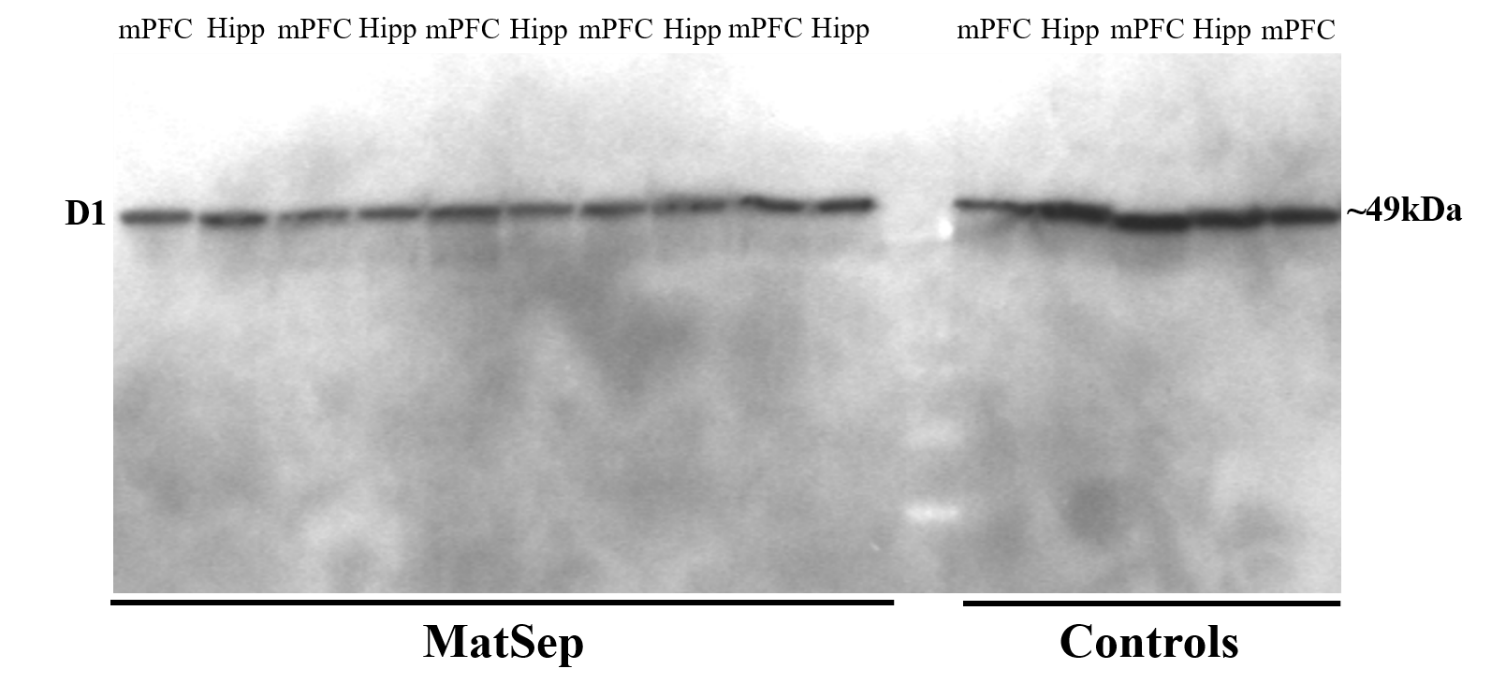


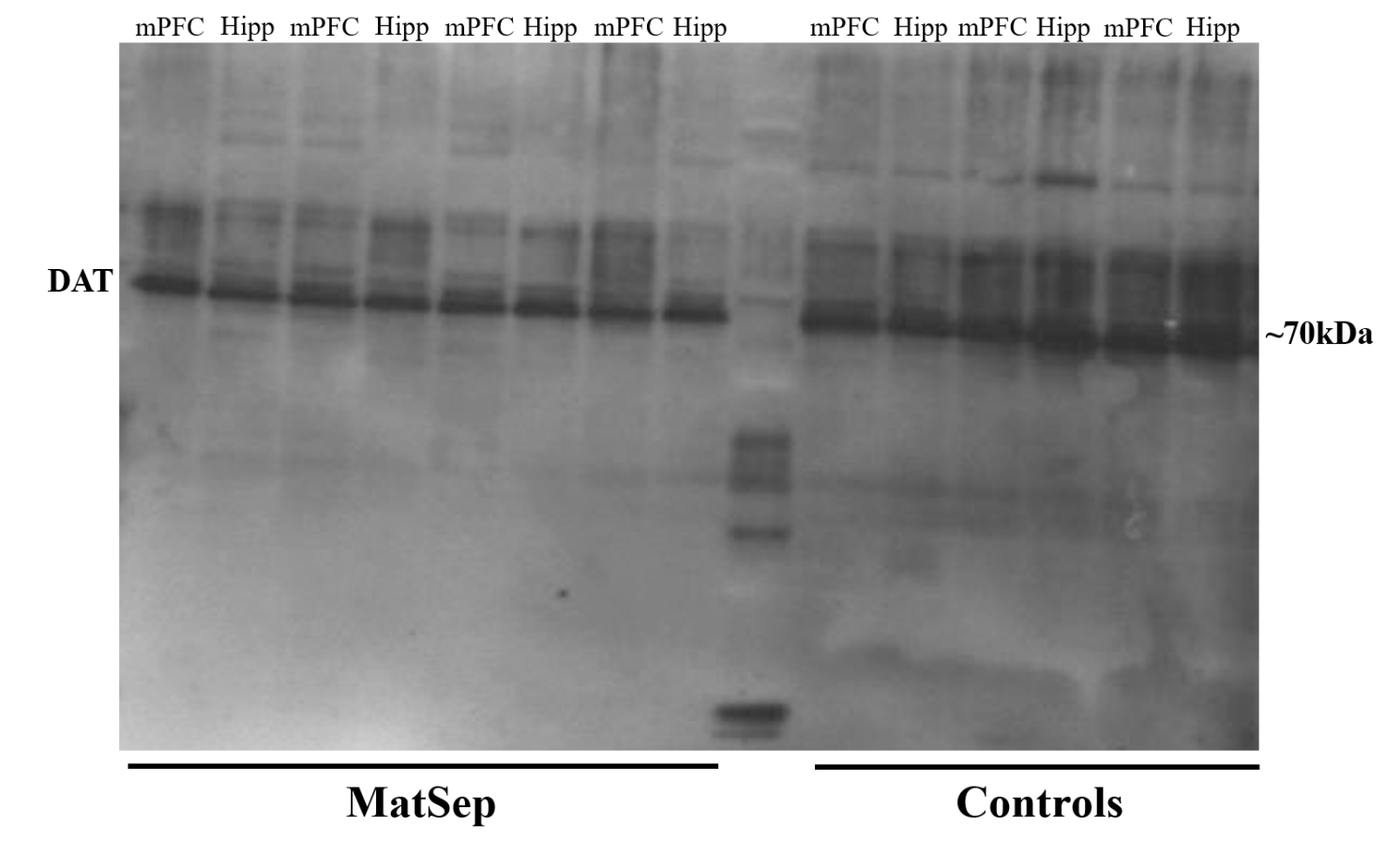


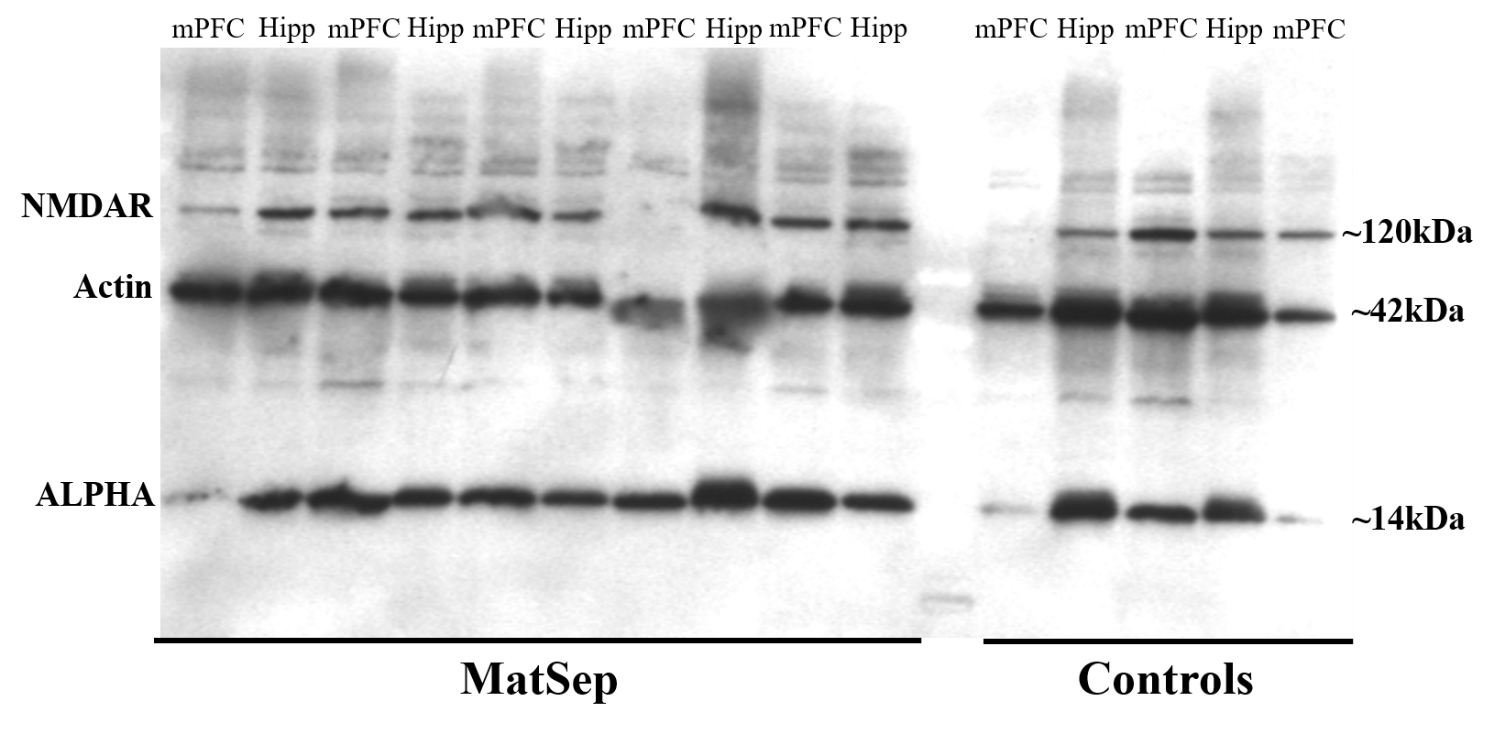


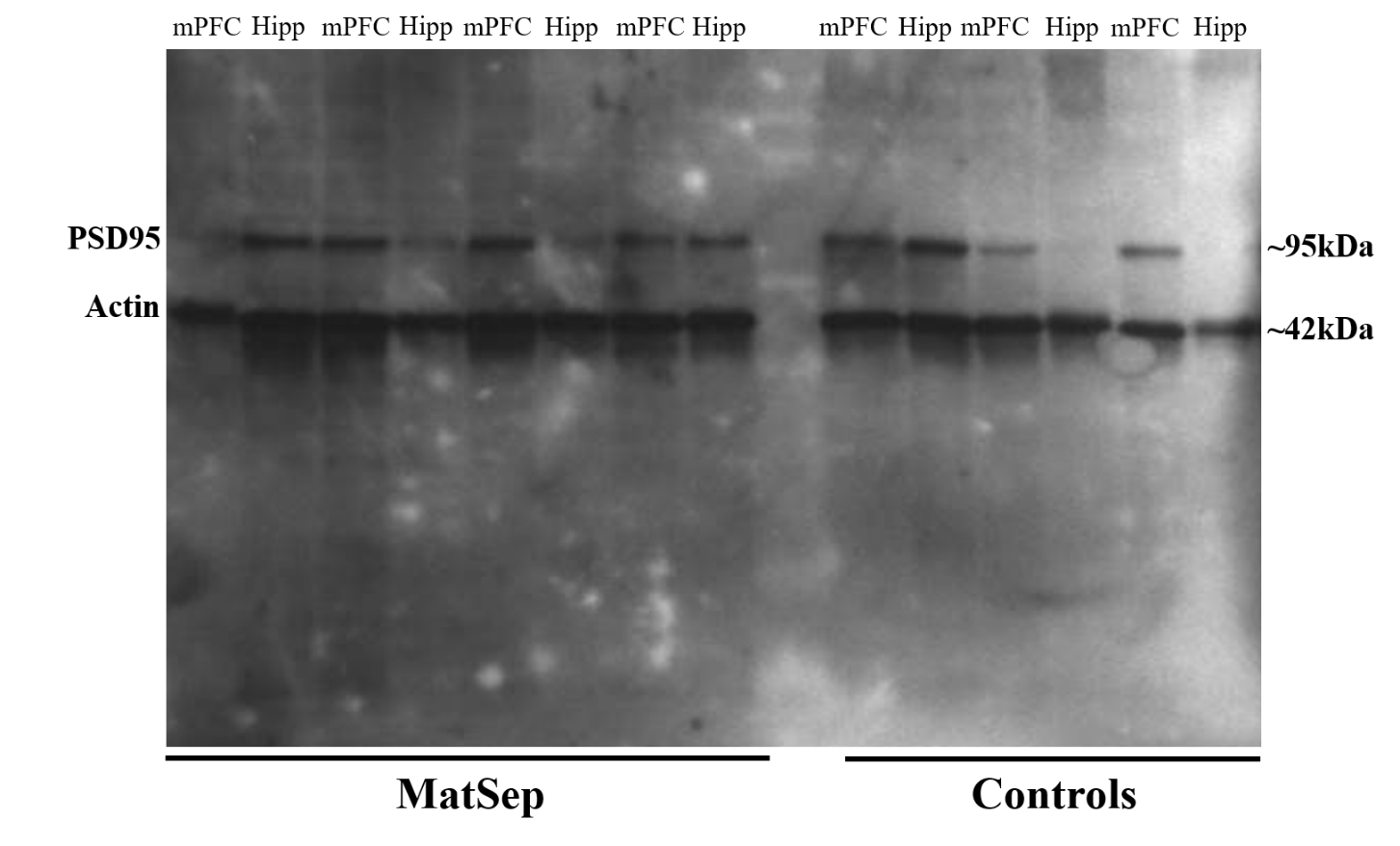


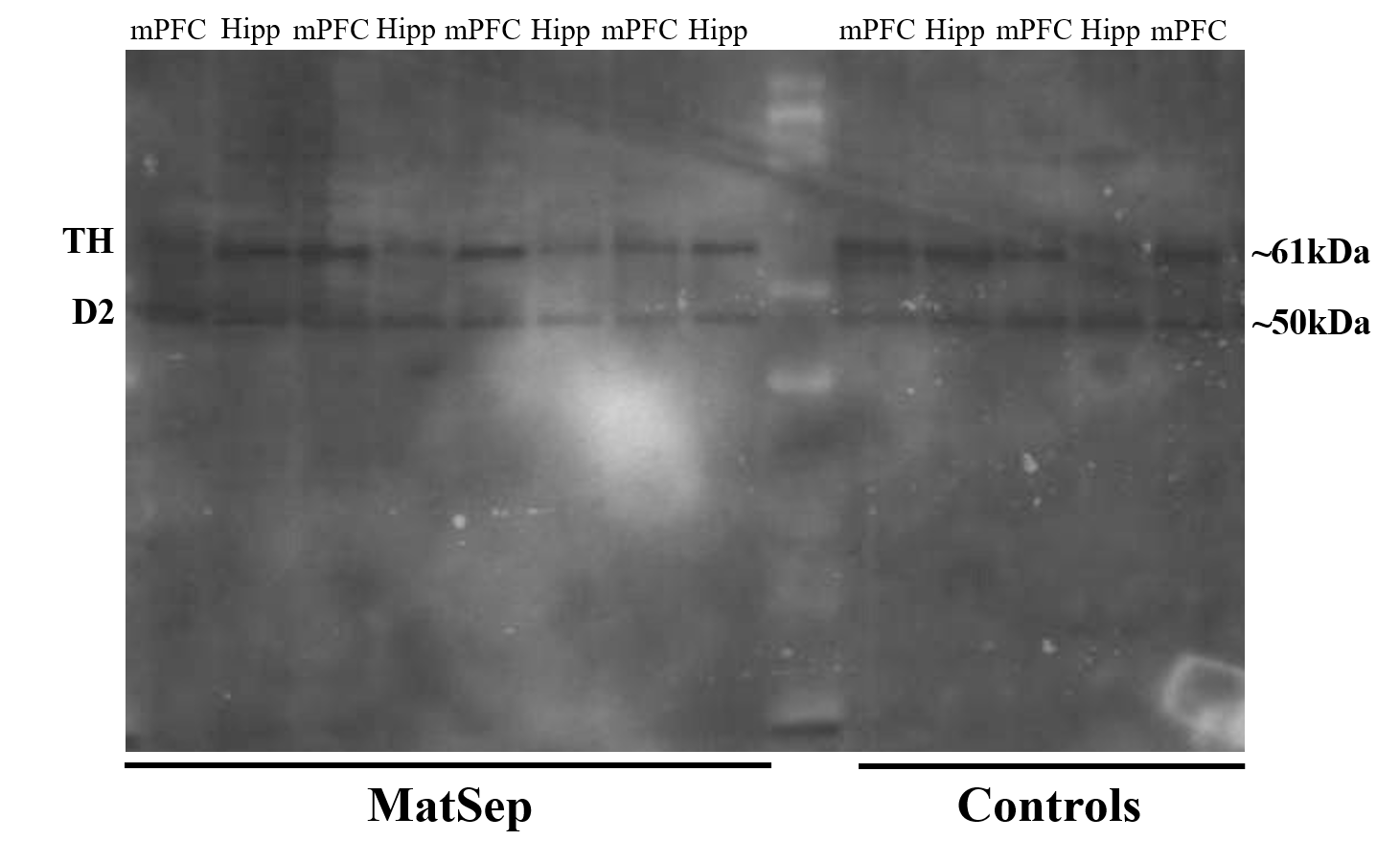


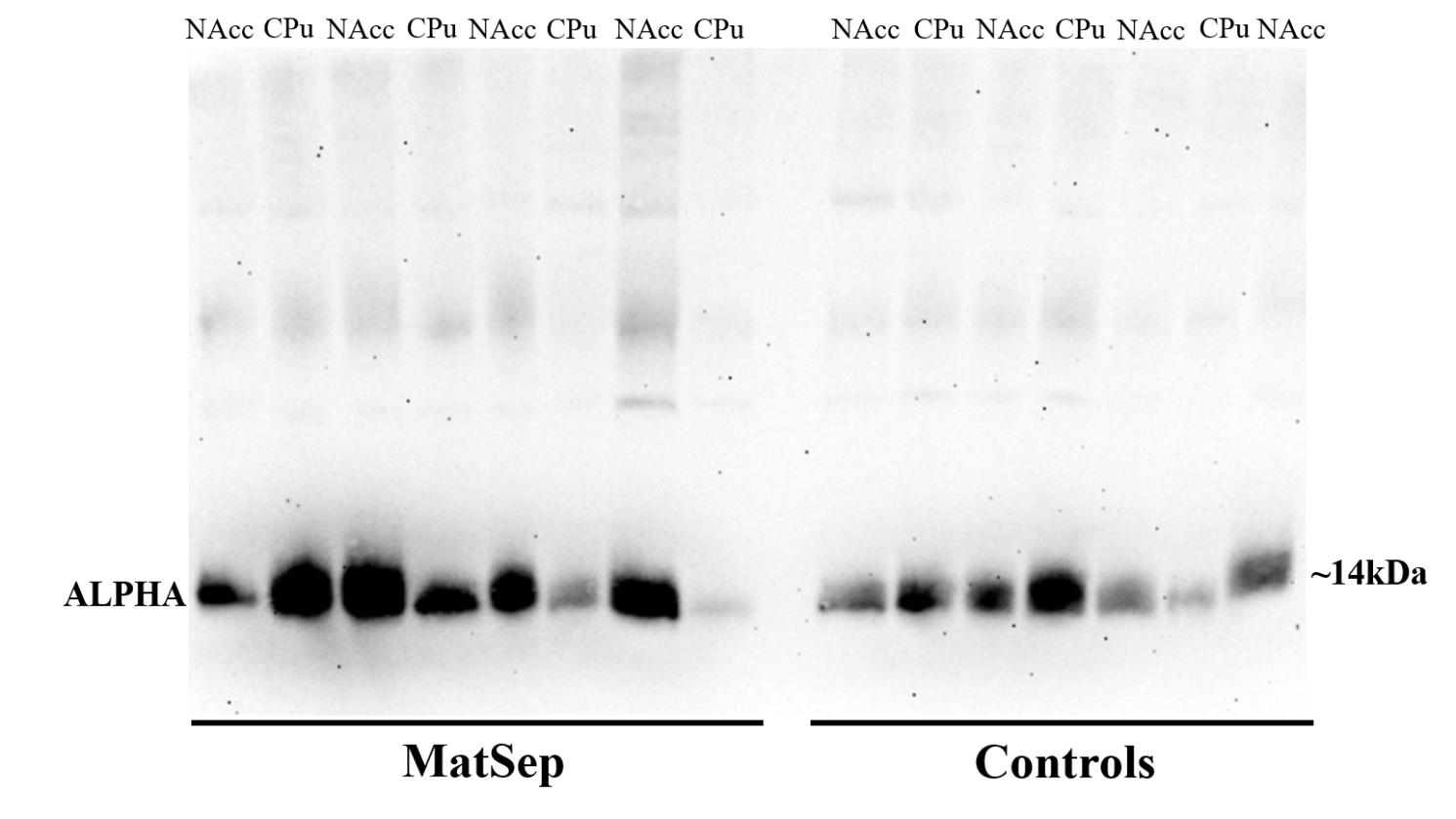


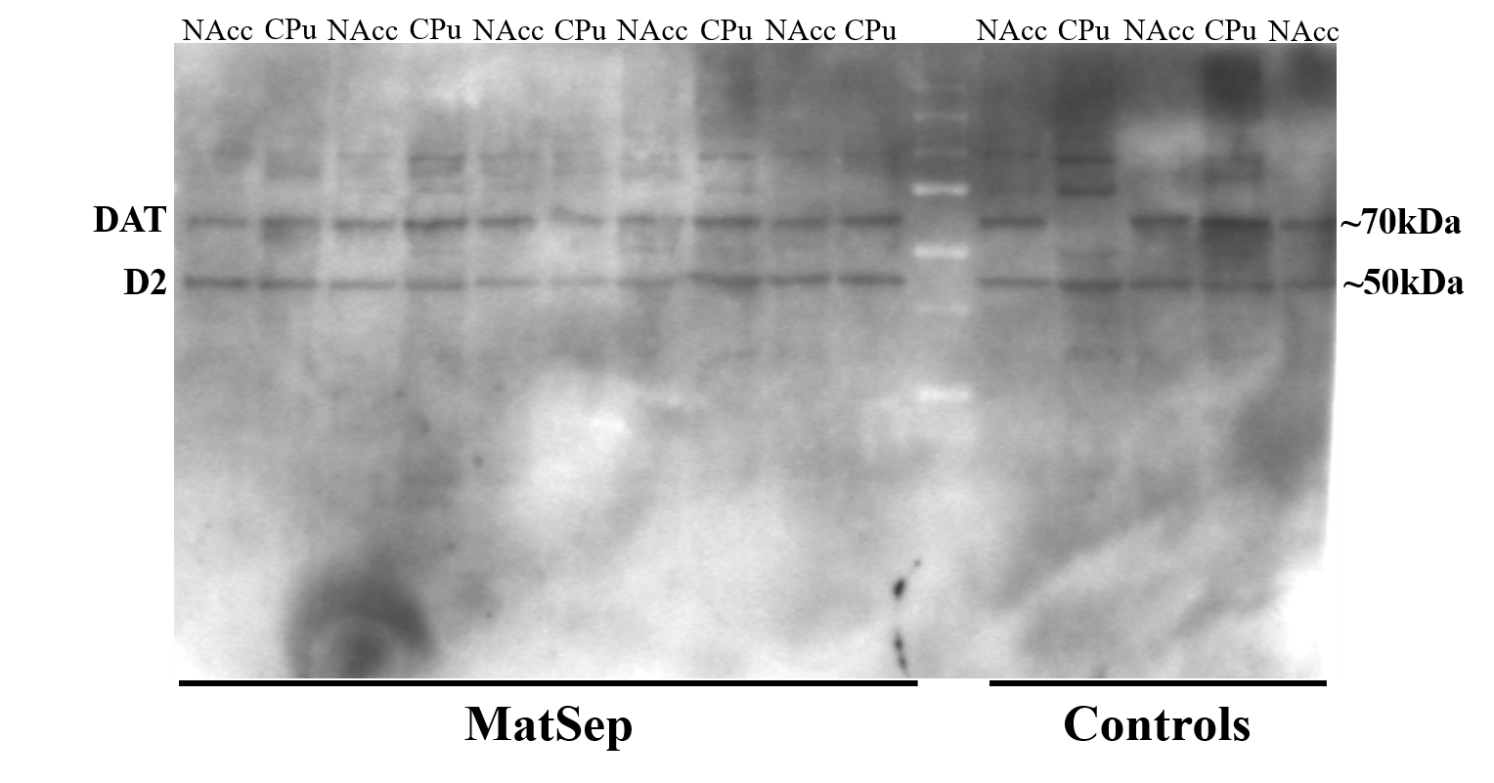


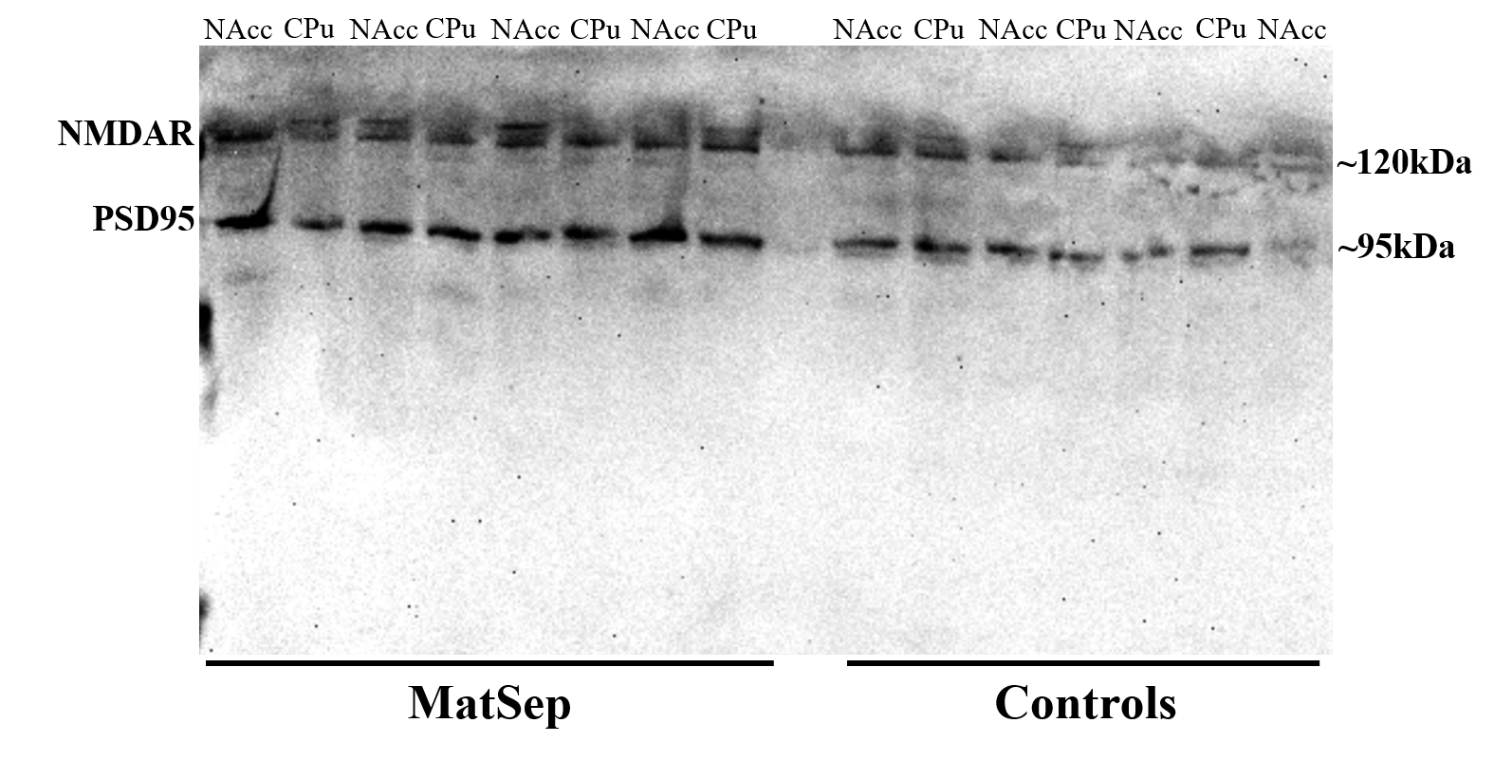


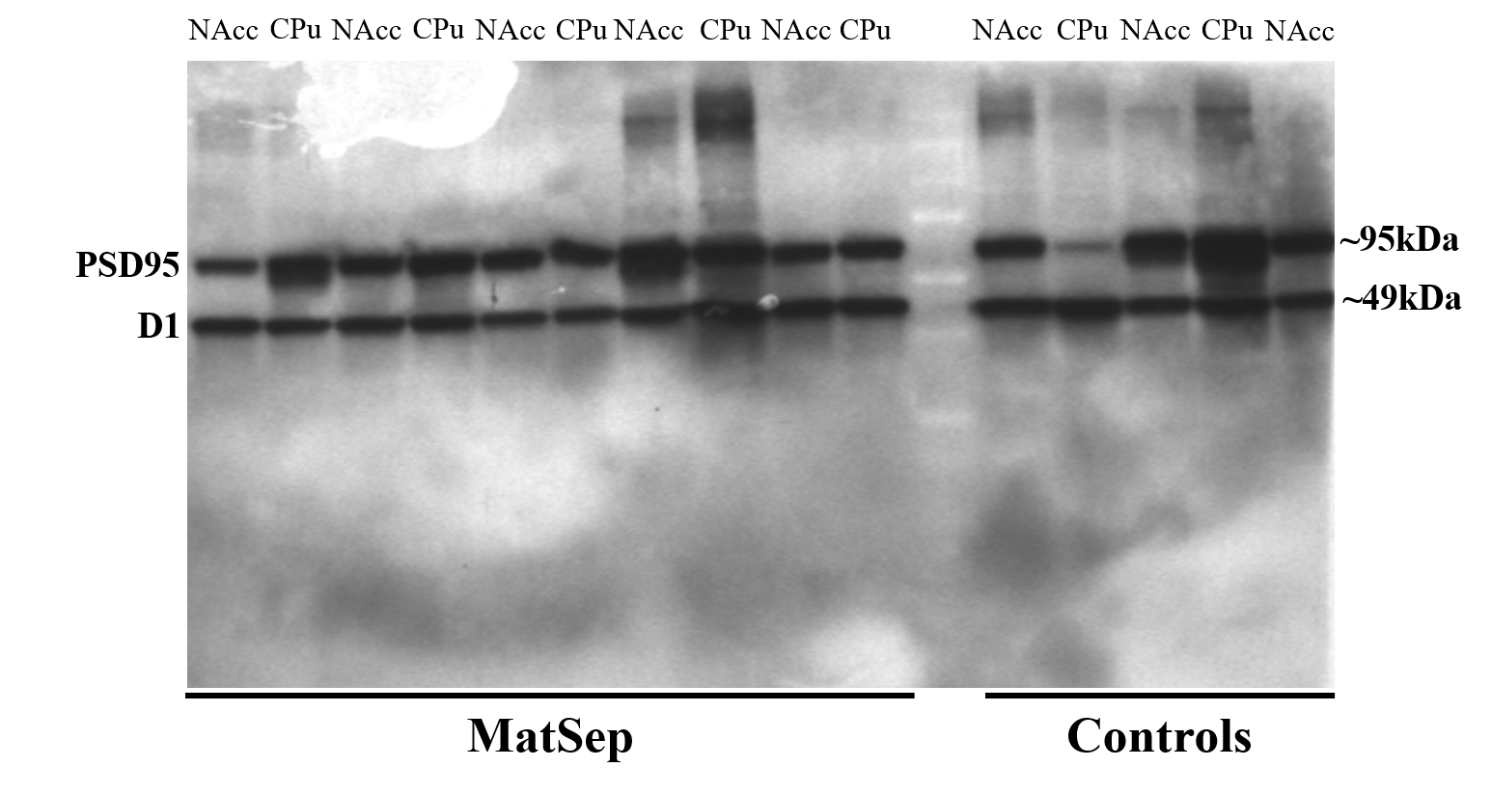


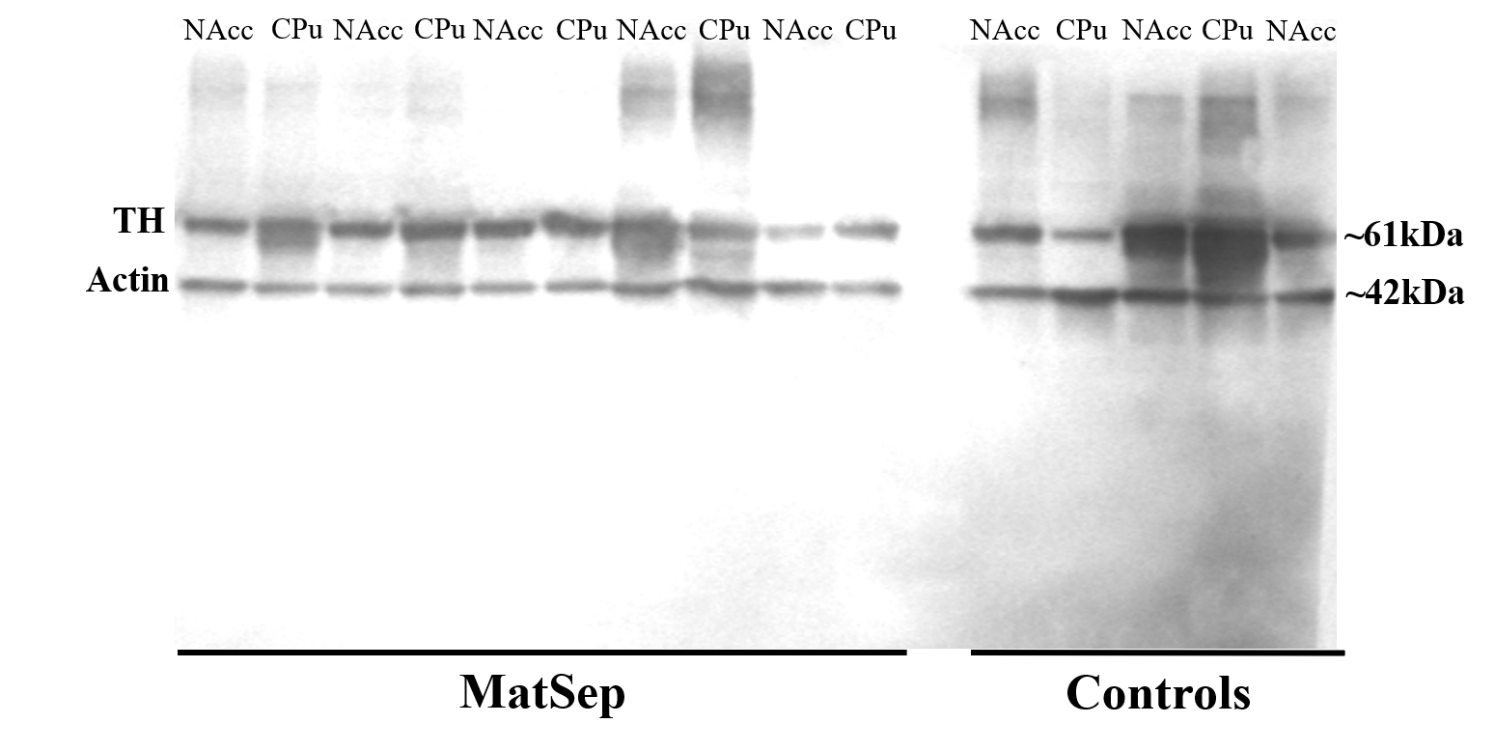


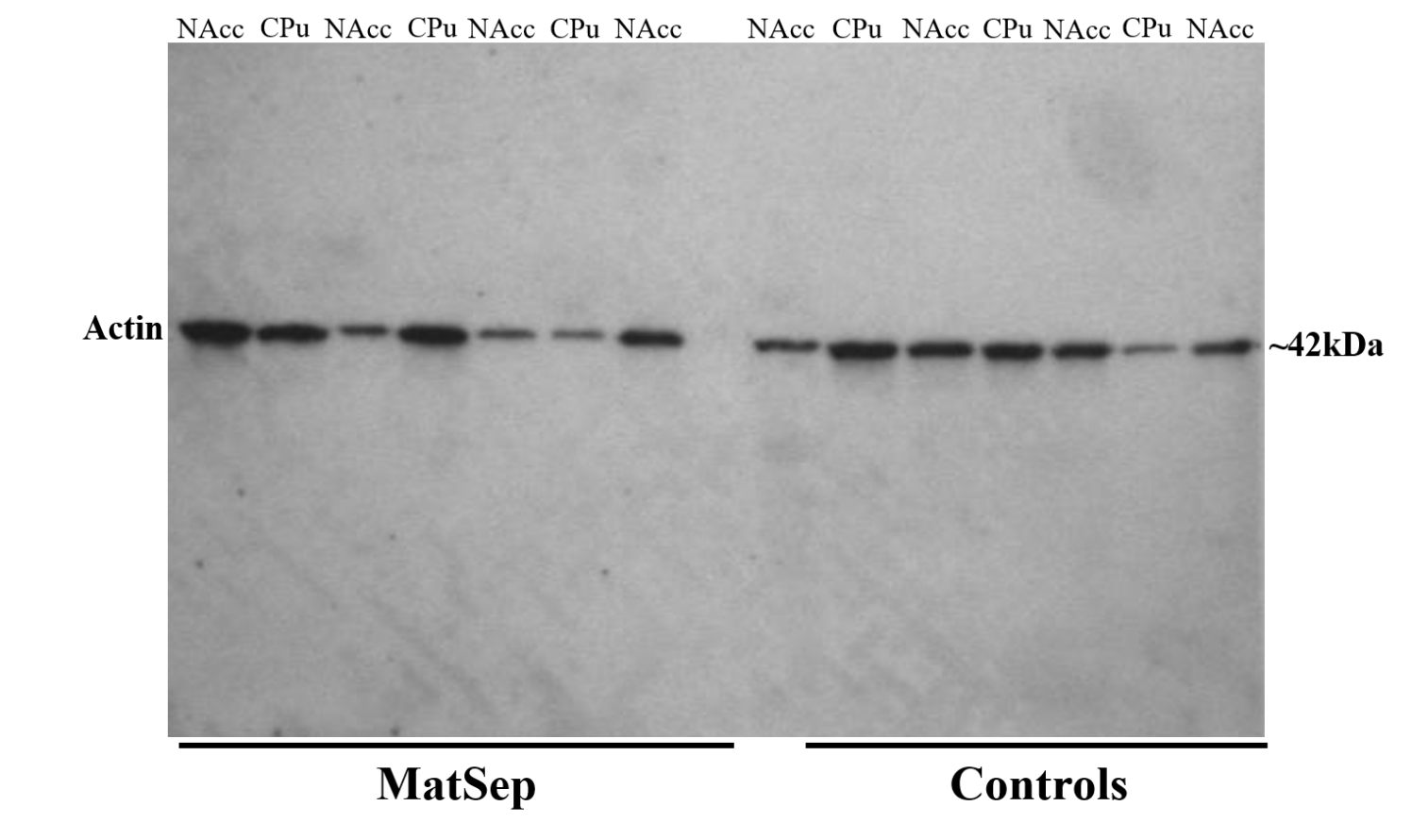

Supplement: Supplementary file 2 [file Data_Sheet_1.docx]
